# The effects of 8 weeks of inspiratory muscle training on the balance of healthy older adults: a randomized, double‐blind, placebo‐controlled study

**DOI:** 10.14814/phy2.14076

**Published:** 2019-05-09

**Authors:** Francesco V. Ferraro, James P. Gavin, Tom Wainwright, Alison McConnell

**Affiliations:** ^1^ Department of Human Sciences and Public Health Bournemouth University Bournemouth United Kingdom; ^2^ School of Health Sciences University of Southampton Southampton United Kingdom; ^3^ Orthopaedic Research Institute Bournemouth University Bournemouth United Kingdom

**Keywords:** Breathing exercise, falls prevention, frail elderly, mini‐BEST, postural balance

## Abstract

To examine the effects of 8‐week unsupervised, home‐based inspiratory muscle training (IMT) on the balance and physical performance of healthy older adults. Fifty‐nine participants (74 ± 6 years) were assigned randomly in a double‐blinded fashion to either IMT or sham‐IMT, using a pressure threshold loading device. The IMT group performed 30‐breath twice daily at ~50% of maximal inspiratory pressure (MIP). The sham‐IMT group performed 60‐breaths once daily at ~15% MIP; training was home‐based and unsupervised, with adherence self‐reported through training diaries. Respiratory outcomes were assessed pre‐ and postintervention, including forced vital capacity, forced expiratory volume, peak inspiratory flow rate (PIFR), MIP, and inspiratory peak power. Balance and physical performance outcomes were measured using the shortened version of the Balance Evaluation System test (mini‐BEST), Biodex^®^ postural stability test, timed up and go, five sit‐to‐stand, isometric “sit‐up” and Biering–Sørensen tests. Between‐group effects were examined using two‐way repeated measures ANOVA, with Bonferroni correction. After 8‐week, the IMT group demonstrated greater improvements (*P* ≤ 0.05) in: PIFR (IMT = 0.9 ± 0.3 L sec^−1^; sham‐IMT = 0.3 L sec^−1^); mini‐BEST (IMT = 3.7 ± 1.3; sham‐IMT = 0.5 ± 0.9) and Biering–Sørensen (IMT = 62.9 ± 6.4 sec; sham‐IMT = 24.3 ± 1.4 sec) tests. The authors concluded that twice daily unsupervised, home‐based IMT is feasible and enhances inspiratory muscle function and balance for community‐dwelling older adults.

## Introduction

Accidental falls are the leading cause of fatal and nonfatal injuries amongst older adults in the Western world (CDCP, 2017). According to the UK's National Institute for Health and Care Excellence, 30% of people over 65 years will fall at least once a year in the UK, whereas 50% of people over 80 years will fall annually (NICE ICGP, 2013). There is an urgent need for effective interventions that are low cost and low risk to reduce falls. The majority of current exercise programs to improve balance focus on lower limb muscle strength, with the addition of supervised multidimensional movements, including Tai Chi, and dance (Sherrington 2019). Recently, it has been suggested that trunk muscle training (i.e., abdominal strength training and Pilates exercises training) may improve balance, and thus be used as a falls prevention intervention for older adults (Granacher et al. 2013). However, the contribution of the trunk muscles to balance is unclear.

Hodges and colleagues (2005) investigated the stabilizing action of the diaphragm muscle, proposing that it works both indirectly, by increasing intraabdominal pressure to support the spine, and directly, by continuous cocontraction contributing in postural stabilization (Hodges and Gandevia 2000). Ageing is associated with biological changes (e.g., senile emphysema) that compromise inspiratory muscle function (Britto et al. 2009) and lung structure (Martin 2010). In particular, the strength of the inspiratory muscles has been reported to decline gradually from 65 years of age onwards (Enright et al. 1994). These age‐related declines in respiratory function may directly, and indirectly, alter the contribution of the inspiratory muscles to balance, in accordance with Hodges' theory. In support of this concept, recent evidence suggests that inspiratory muscle weakness may contribute to balance deficits during daily activities, such as chair rising (Janssens et al. 2014). Therefore, this raises the question as to whether inspiratory muscle training (IMT), which consists of breathing exercises using a pressure threshold device can reduce the physiological effect of ageing on inspiratory muscle function, thereby improving balance ability for older adults.

The purpose of this study was to evaluate the effectiveness of 8 weeks IMT on the inspiratory muscle function, balance, and physical performance of community‐dwelling older adults. Based on the aforementioned findings of Hodges and colleagues, we hypothesize that 8 weeks of twice daily home‐based, unsupervised IMT will improve inspiratory muscle function and balance performance, without any adverse events (i.e., falls accident).

## Methods

### Participant characteristics

Fifty‐nine (18 male) community‐dwelling older adults (74 ± 6 years old) volunteered to take part. Exclusion criteria comprised: aged under 65 years, chronic lung condition (e.g., asthma, obstructive pulmonary disease), moderate or severe low back pain (Oswestry low back pain [ODI] questionnaire higher than 21%) (Fairbank and Pynsent 2000), cognitive impairments (Mini–Mental State Examination [MMSE] score lower than 24) (Folstein et al. 1983), fear of falling (Activities Balance Confidence [ABC] scale lower than 67%) (Powell and Myers 1995), having fallen in the previous 24 months, diabetes (Allet et al. 2008), heart conditions preventing physical activity, taking beta‐blocker medication, vertigo in the past 6 months, currently undertaking exercise balance training (including Tai Chi and Pilates) and any experience of IMT (as participants would have been able to recognize the sham‐IMT). These were assessed through completion of a health check questionnaire as part of the selection process.

Recruitment occurred via Bournemouth University public engagement events, and participants gave written informed consent before taking part. Participants met with the principal investigator (FVF) on two occasions: firstly, at baseline (week 1), and secondly, postintervention (week 8). The research protocol was approved by Bournemouth University Research Ethics Committee (Reference ID: 15352).

### General design

**Figure 1:**
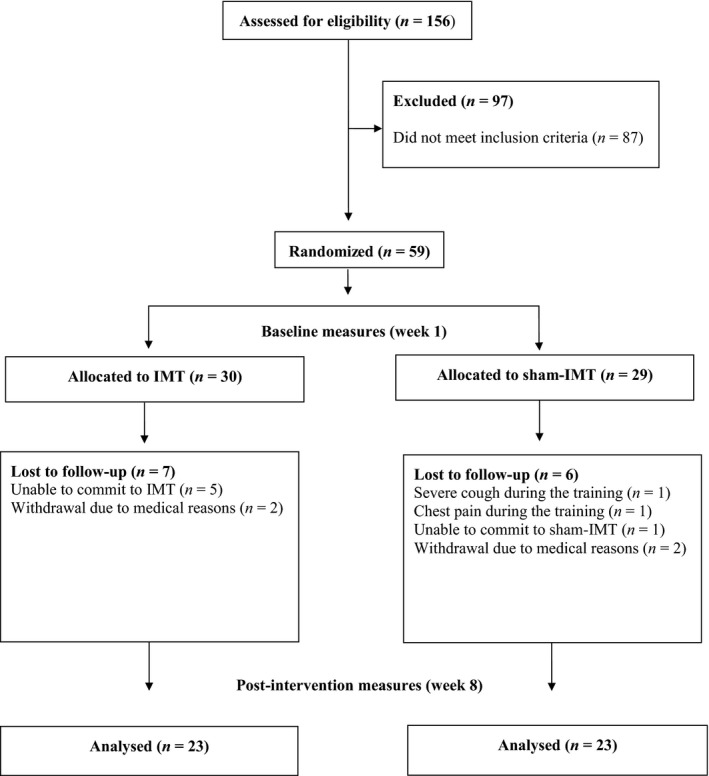
PRISMA flow diagram displaying participant pathways through the study. IMT = inspiratory muscle training.

A double‐blind, randomized placebo‐controlled design was used, with participants allocated randomly to undergo either IMT or sham‐IMT. An equal number of POWERbreathe^®^ Medic Plus devices were prepared and assigned to each participant randomly, using a pseudorandom number generator (randomizer.org) by simple randomization, without stratification on gender. Figure 1 presents details of screening, sample size, group allocation, and withdrawals.

All measurements were performed between June 2017 and February 2018 at the Orthopaedic Research Institute (Bournemouth, UK) under standardized conditions, in a temperature‐controlled laboratory (20–22°C; humidity < 70%). Testing and re‐testing sessions were scheduled at a similar time of the day (between 9:00 and 11:00 am) to minimize potential effects of diurnal variation (Atkinson and Reilly 1996). Participants were advised not to consume caffeine, alcohol, or to perform strenuous exercise 2 h before measurements. Assessments of forced vital capacity (FVC), forced expiratory volume in 1 sec (FEV_1_), peak inspiratory flow rate (PIFR), maximal inspiratory pressure (MIP), peak inspiratory power (PP), balance proficiency measured with the shortened version of the Balance Evaluation System test (mini‐BEST) and the Biodex^®^ postural stability tests, physical performance (five sit‐to‐stand and timed up and go tests), and trunk muscle endurance (sit‐up and Biering–Sørensen tests) were performed pre‐ and postintervention, by one researcher (FVF) blinded to group allocation.'

### Procedures

#### Pulmonary and inspiratory muscle function

##### Pulmonary function

A spirometer (SpiroUSB, Care Fusion, Wokingham, Berkshire, UK) was used to measure FVC and FEV_1_, following the guidelines of the American Thoracic Society (Miller et al. 2005), whilst PIFR was measured using the POWERbreathe^®^ K5, with Breathe‐Link 2.0 software (POWERbreathe^®^ International Ltd, Southam, UK) (Langer et al. 2013). Participants performed forced breathing maneuvres while wearing a nose clip, at least five, and no more than eight times until variability was within 5% for three consecutive maneuvres and the highest score was recorded.

##### Respiratory muscle function

A hand‐held mouth pressure meter (MicroRPM, Micro Medical Ltd, Rochester, Kent, UK) was used to determine MIP. The pressure meter was fitted with a side port opening of 1 mm internal diameter, to maintain glottis opening. All participants practised the Müller maneuvre three times before testing, and MIP measurements were repeated, at least five, and no more than eight times, until variability was within 10% for three consecutive maneuvres.

Inspiratory peak power (PP) was measured using the POWERbreathe^®^ K5 with Breathe‐Link 2.0 software (Langer et al. 2013). Inspiratory muscle power analysis was undertaken at six different loads (40, 50, 55, 60, 70, and 80% of participants` baseline MIP) and data were fitted in a polynomial curve, replicating methods reported elsewhere (Romer and McConnell 2003). Participants were requested to inhale with maximal effort against the six loads, the order of which was randomly assigned (using randomizer.org software). Pressure and flow measurement were then obtained for each percentage of baseline MIP, and inspiratory muscle power was calculated from the instantaneous product of inspiratory pressure and flow.

Three trials were performed for each of the loading intensities with 30 sec rest between efforts, making a total of 18 forced inspiratory maneuvres. For all pulmonary and respiratory muscle tests, a nose clip was worn, and verbal encouragement was provided to promote maximal efforts. In each of the two respiratory muscle function measurements, the highest was recorded.

### Physical performance

#### The five sit‐to‐stand test

The five sit‐to‐stand test (FSTS) involved measuring the time taken to stand from a seated position five consecutive times (Watt et al. 2018). Briefly, participants were asked to sit on the edge of an armless chair (sitting height 46 cm, seat length 45 cm) with their arms folded across their chest. They were instructed to rise, and then become seated as fast as possible, five times, with both feet maintaining contact with the floor. Timing commenced on the command “3, 2, 1 and go”. The FSTS acted as a proxy measure of lower‐limb strength and an outcome measure.

The preactivation five sit‐to‐stand (FSTS_PA_) was used to determine the effect of acute, preinspiratory muscle activation (Volianitis et al. 2001) on the FSTS task. Briefly, rest intervals between FSTS and FSTS_PA_ were provided (2–5 min, consistent pre‐ and postintervention), when ready, participants performed 30 repetitions of forceful inhalations against a load equivalent to ~40% of their MIP, followed by forceful inhalation at 80% of MIP until repetition failure. Failure was indicated by three successive unsuccessful attempts to inhale against the load. Verbal encouragement was provided during the 80% loading phase by the principal investigator (FVF) at baseline and postintervention.

The preactivation session was performed with Breathe‐Link software so that it was possible to look at each attempt and detect when there was no more passage of air through the device (i.e., participant was exhausted and unable to perform inhalation at 80% of baseline MIP). When participants were unable to inhale forcefully for three consecutive attempts, they were instructed to stop the forced inspiration and to perform the FSTS_PA_. During both FSTS and FSTS_PA_ tests, participants were blindfolded, to minimize a potential effect of vision on the performance (Mourey et al. 2000).

#### The timed up and go test

The timed up and go test (TUG) was undertaken to assess mobility and gait speed, in single and dual‐task condition, i.e., cognitive timed up and go (TUG_C_) and motor timed up and go (TUG_M_). For all TUG conditions, participants were asked to sit on the edge of an armless chair (sitting height 46 cm). On the command “3, 2, 1 and go” they were instructed to stand up, walk at their habitual pace to a line on the floor 3 m away, turn around, walk back and sit down (Podsiadlo and Richardson 1991). During the TUG_C_, participants were instructed to perform the same task while counting aloud, backwards in threes from a randomly selected number between 80 and 100. During the TUG_M_ participants completed the same tasks, as in the TUG, while holding a drinking glass (diameter 8 cm, height 9.5 cm) filled with water (1 cm away from the edge of glass). Before testing, a familiarization trial of the TUG task was performed. The three tasks were undertaken in random order, and no physical assistance was provided.

### Balance

#### Mini‐BEST test

Static and dynamic balances were assessed using the mini‐BEST, which included 14 different tasks, divided into: anticipatory (e.g., toe rise for 3 sec), reactive postural control (e.g., compensatory stepping forward), sensory orientation (e.g., stand on a foam surface with eyes closed for 30 sec), and dynamic gait (e.g., walk with horizontal head turns) (O'Hoski et al. 2015).

#### Biodex^®^ postural stability test

Static balance was further investigated using the Biodex^®^ postural stability test (PST) (Biodex, Shirley, NY) (Parraca et al. 2011). The PST measured participants' ability to maintain their center of pressure within their base of support, with a lower score indicating a better balance. Participants were asked to step blindfolded onto a firm, stable surface, as well as an unstable foam surface, three times, each for 20 sec. This test was also repeated after inspiratory muscle preactivation following the same protocol described for the FSTS_PA_. The latter tests are reported as stable PST_PA_ and unstable PST_PA_, respectively.

### Trunk muscle endurance

Anterior trunk muscle endurance was assessed using an isometric “sit‐up” task, adopting a bent knee sit‐up, with feet secured by a strap, arms folded across the chest and trunk forming an angle with the horizontal of 60° (McGill et al. 1999). Posterior trunk muscle endurance was assessed using the Biering–Sørensen test, where participants were asked to maintain a prone position, facing the floor, with their torso unsupported over the edge of a test bench. A strap secured their legs and hips, and hands were placed behind their head (Biering‐Sørensen 1984). Participants were instructed to hold the static positions until volitional exhaustion, without verbal encouragement. Time was recorded using a stopwatch.

### Interventions

Both the research team and participants were blinded to group allocation, and participants were unaware of the predicted outcomes of the two interventions. To preserve intervention blinding, motivational telephone calls were not provided during either intervention. Adherence to the prescribed training was self‐reported through weekly training diaries by both groups, which were presented to the researcher postintervention, after the follow‐up assessments (week 8).

#### Inspiratory muscle training

Participants performed home‐based IMT twice daily [morning: between 7:00 and 12:00 and evening: between 16:00 and 21:00], for 8 consecutive weeks, using a mechanical pressure threshold loading device (POWERbreathe Plus, POWERbreathe^®^ International Ltd, Southam, UK). Participants followed an established training protocol known to improve inspiratory muscle function, consisting of 30 quick breaths twice a daily at an adjustable resistance (equivalent to ~50% of baseline MIP). In addition, participants in this group were able to increase the inspiratory resistance when they felt that 30 breaths were achievable with ease or if they could reach 35 consecutive breathes (McConnell 2013).

#### Sham – inspiratory muscle training

Participants performed 60 slow breaths once daily at a load setting of 0 (corresponding to ~15% baseline MIP), using the same device as the IMT group. For the sham group, the ability to adjust the training load was prevented using sticky tape applied to the device's load adjuster. This protocol has been shown previously to elicit negligible changes in inspiratory muscle function in healthy young adults (Romer et al. 2002) and in those with chronic obstructive pulmonary disease (Charususin et al. 2018).

### Data analysis

Sample size estimation was made using G*Power software (Faul et al. 2007), using data from a pilot study involving 33 participants (73 ± 6 years) (unpublished) with an *α* error = 0.05 and 1 − *β = 0*.95. Briefly, 26 participants completed 8 weeks of unsupervised, home‐based IMT. Measured of balance (assessed with the mini‐BEST) pre‐ and postintervention showed a significant improvement from 21.2/28.0 ± 3.8 to 24.1/28.0 ± 2.1 (13.7%, *P* < 0.01, *d* = 0.8).

To assess the hypothesis that IMT improves dynamic balance performance (measured with the mini‐BEST) a total sample of 46 participants were required to demonstrate an improved of 10%. The protocol analysis was used to examine between‐group intervention (IMT vs. sham‐IMT), pre‐ versus postintervention. Further within‐group effects were explored using a paired *t*‐test and a Wilcoxon signed ranks test for not normally distributed data.

Between‐group comparisons were made using a two‐way repeated‐measures ANOVA, with Bonferroni. Data are reported as mean, standard deviation (SD), and percentage change. Statistical significance between‐group (intervention x time interaction) was determined a priori as *P* ≤ 0.05 and Cohen's *d* effect sizes were calculated to determine the effect magnitude (small *d *≤* *0.2; medium 0.2 < *d *≤* *0.8; large *d *>* *0.8).

## Results

Forty‐six participants completed the study; training adherence was 76% and 79% for IMT and sham‐IMT, respectively. Following the interventions, there were no adverse events (i.e., fall accidents or respiratory impairments) in the IMT group, whereas two participants reported severe cough and chest discomfort during sham‐IMT. Reasons for withdrawing are reported in Figure 1. Groups were similar in gender, age, BMI, pulmonary function (FVC and FEV_1_), balance confidence (ABC), perception of back pain (ODI), and cognitive capacity (MMSE) before training (*P* > 0.05). See Table 1.

**Table 1:** Participant characteristics. Pulmonary and inspiratory muscle function tests at baseline and postintervention

Outcomes	IMT (*n* = 23)			Sham‐IMT (*n* = 23)			*P*‐values
	Baseline	Postintervention	% change	Baseline	Postintervention	% change	Between‐groups
Gender (M/F)		9/14			9/14		N/A
Age (years)		75 ± 6			72 ± 5		N/A
BMI (kg m^−2^)		27 ± 3.1			26 ± 3.5		N/A
ABC	90.5 ± 7.2	91.7 ± 9.5	1.3	84.9 ± 12.8	86.7 ± 10.4	2.1	NS
ODI	3.5 ± 5.7	3.3 ± 5.1	−5.7	4.6 ± 5.7	3.8 ± 4.1	−17.4	NS
MMSE	28 ± 1.6	29 ± 0.9	3.5	28 ± 0.8	29 ± 0.9	3.5	NS
FVC (L)	3.4 ± 0.9	3.5 ± 0.7	2.9	3.2 ± 0.8	3.3 ± 0.8	3.1	NS
FEV_1_ (L sec^−1^)	2.6 ± 0.7	2.6 ± 0.9	0	2.3 ± 0.6	2.3 ± 0.6	0	NS
PIFR (L sec^−1^)	4.6 ± 0.9	5.5 ± 0.6**	19.7	4.0 ± 1.4	4.3 ± 1.4*	7.5	*P* = 0.02
MIP (cmH_2_O)	76.0 ± 27.4	110.9 ± 21.3**	45.9	72.8 ± 40.9	85.9 ± 28.8*	18.0	NS

BMI, body mass index; ABC, activities specific balance confidence scale; ODI, Oswestry low back pain disability questionnaire; MMSE, mini‐mental examination test; FVC, forced vital capacity; FEV_1_, forced expiratory volume in 1 sec; PIFR, peak inspiratory flow rate; MIP, maximal inspiratory pressure. N/A, not applicable; NS, not significant. ^*^Significantly different from baseline (*P* ≤ 0.05), ^**^significantly different from baseline (*P* ≤ 0.01).

### Pulmonary function

Forced vital capacity and FEV_1_ were not significantly different following either intervention (Table 1), whereas both groups improved PIFR postintervention, with a significant effect between‐groups (*P* = 0.02). Specifically, within‐participants analysis showed that IMT increased PIFR by 19.7% (*d *=* *1.2; *P* < 0.01), whilst sham‐IMT increased PIFR by 7.5% (*d *=* *0.2; *P* = 0.05).

### Inspiratory muscle function

**Figure 2:**
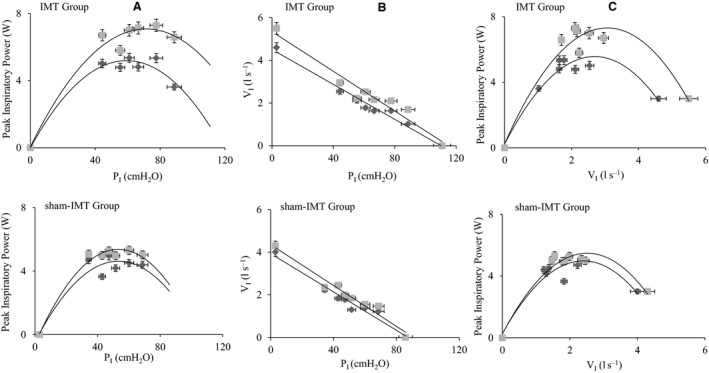
(A) Peak inspiratory power (Watts) versus inspiratory load pressure P_I_ (cmH_2_O). (B) Inspiratory flow rate V_I_ (L sec^−1^) versus Inspiratory mouth pressure P_I_ (cmH_2_O). (C) Peak inspiratory power (Watts) versus inspiratory flow rate V_I_ (L sec^−1^). Before (■) and after (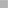) 8 weeks of inspiratory muscle training (IMT) and sham‐IMT. Data are represented in both axes as mean ± percentage error.

Both groups showed significant improvements in MIP postintervention (Table 1), with no significant changes between groups. The IMT group increased MIP by 45.9% (*d *=* *1.4; *P* ≤ 0.01), and the sham‐IMT group increased by 18.0% (*d *=* *0.3; *P* = 0.02). Changes in inspiratory peak power (PP) were not significant between‐groups (Table 2). However, within‐participant analysis revealed significant improvements in PP at all loads for the IMT group, with the highest inspiratory power improvement (80.6%; *d* = 0.8; *P* = 0.008) at the 80% MIP load. In contrast, following sham‐IMT, inspiratory peak power increased significantly only at the 80% MIP load (16.3%; *d *=* *0.02; *P* = 0.03). Figure 2 presents the interrelationships of the inspiratory loads applied, inspiratory peak power, and peak inspiratory flow rates.

**Table 2:** Baseline and postintervention values for peak inspiratory power at different percentages of load

Outcomes	IMT (*n* = 21)			Sham‐IMT (*n* = 22)			*P*‐values
	Baseline	Postintervention	% change	Baseline	Postintervention	% change	Between‐groups
PP at 40% MIP (W)	5.0 ± 2.0	6.7 ± 3.1*	34.0	4.7 ± 3.5	5.1 ± 3.9	8.5	NS
PP at 50% MIP (W)	4.7 ± 2.7	5.7 ± 2.7*	21.3	3.6 ± 3.1	4.9 ± 3.7	36.1	NS
PP at 55% MIP (W)	5.3 ± 3.1	7.0 ± 3.2*	32.1	4.9 ± 3.9	5.2 ± 3.4	6.1	NS
PP at 60% MIP (W)	4.8 ± 2.4	7.1 ± 3.7**	47.9	4.1 ± 3.5	4.9 ± 2.9	19.5	NS
PP at 70% MIP (W)	5.3 ± 3.4	7.3 ± 3.9*	37.7	4.5 ± 4.1	5.3 ± 3.5	17.8	NS
PP at 80% MIP (W)	3.6 ± 2.4	6.5 ± 4.3**	80.6	4.3 ± 3.1	5.0 ± 4.4*	16.3	NS

PP, peak inspiratory power, MIP, maximal inspiratory pressure; W, Watts; NS, not significant.

* Significantly different from baseline (*P* ≤ 0.05).

** Significantly different from baseline (*P* ≤ 0.01).

### Physical performance

For the FSTS and FSTS_PA_ tasks (Table 3), there were no significant between‐group differences following the interventions. However, there were significant within‐participant changes for FSTS_PA_ in the sham‐IMT group, which needed 20.5% more time to complete the task (*d *=* *0.5; *P* = 0.03). Postintervention, both groups were significantly faster in the FSTS_PA,_ than the FSTS test. In particular, time decreased by 10.7% in IMT group (*d *=* *0.2; *P* ≤ 0.01) and by 10.0% in sham‐IMT (*d *=* *0.2; *P* ≤ 0.01). The postintervention changes in TUG, TUG_C_, and TUG_M_, were significantly different between the groups (*P* = 0.03; *P* = 0.02; *P* = 0.02, respectively). The TUG was also significantly different within the IMT participants, who needed 5.3% less time to complete the task (*d *=* *0.2; *P* = 0.04). Further analysis between single and dual‐task conditions at baseline and postintervention showed that cognitive (TUG_C_) and motor (TUG_M_) dual tasks were both significantly different (*P* ≤ 0.01) from the single‐task (TUG) condition at baseline and postintervention in IMT and sham‐IMT groups. However, the data also showed that changes in motor dual task were significantly different from changes in cognitive dual‐task (*d *=* *0.4; *P* ≤ 0.01) postintervention in sham‐IMT group and not in the IMT group.

**Table 3:** Baseline and postintervention values for physical performance, Biodex^®^ postural stability index and trunk endurance tests

Outcomes	IMT (*n* = 23)			Sham‐IMT (*n* = 23)			*P*‐values
	Baseline	Postintervention	% change	Baseline	Postintervention	% change	Between‐groups
FSTS (s)	15.5 ± 5.9	16.8 ± 8.1	8.4	18.6 ± 11.5	20.9 ± 8.9	12.4	NS
FSTS_PA_ (s)	15.1 ± 5.9	15.0 ± 5.8††	−0.7	15.6 ± 4.9	18.8 ± 7.5^A^, ††	20.5	NS
TUG (s)	7.6 ± 1.6	7.2 ± 1.8 A	−5.3	9.0 ± 2.4	9.3 ± 3.6	3.3	*P* = 0.03
TUG_C_ (s)	10.0 ± 3.0**	10.3 ± 5.1 ##	3.0	12.3 ± 3.8**	13.5 ± 4.7 ##	1.6	*P* = 0.02
TUG_M_ (s)	10.3 ± 2.2**	9.2 ± 2.2 ##	−10.7	11.7 ± 3.5**	11.8 ± 3.3 ##, ‡‡	0.9	*P* = 0.02
Stable PST	2.8 ± 0.8	2.0 ± 0.7	−27.8	2.7 ± 0.9	1.7 ± 0.7	−35.7	NS
Stable PST_PA_	2.6 ± 0.9	2.4 ± 0.8	−9.5	2.6 ± 0.8	2.2 ± 0.7	−13.2	NS
Unstable PST	3.1 ± 1.4	2.7 ± 1.2	−11.7	3.2 ± 1.4	1.9 ± 1.4	−10.5	NS
Unstable PST_PA_	2.6 ± 1.2	2.8 ± 1.2	6.5	3.0 ± 1.4	2.8 ± 1.4	−7.6	NS
Sit‐up (s)	59.9 ± 14.5	87.2 ± 17.9	45.6	31.8 ± 5.5	39.3 ± 9.8	23.6	*P* = 0.02
Biering–Sørensen (s)	64.7 ± 7.3(*n* = 22)	105.4 ± 13.7^AA^	62.9	37.1 ± 6.7(*n* = 17)	46.1 ± 8.1	24.3	*P* = 0.001

TUG, timed up and go; TUG_C_, cognitive TUG; TUG_M_, motor TUG; FSTS, five sit‐to‐stand; FSTS_PA_, FSTS preinspiratory muscles activation; PST, postural stability index; PST_PA_, PST prior inspiratory muscles activation; s, seconds; NS, not significant.

A Significantly different from baseline (*P* ≤ 0.05).

AA Significantly different from baseline (*P* ≤ 0.01).

†† Significantly different from no preinspiratory muscle activation condition (*P* ≤ 0.01).

** Significantly different from TUG condition at baseline (*P* ≤ 0.01).

## Significantly different from TUG condition postintervention (*P* ≤ 0.01).

‡‡ Significantly different from TUG_C_ condition postintervention (*P* ≤ 0.01).

### Balance

The mini‐BEST score differed within‐participants and between‐groups (*P* = 0.05) postintervention (Table 4). The IMT group improved by 18.1% (*d *=* *1.3; *P* ≤ 0.01), specifically in Reactive (by 39.4%; *d *=* *0.8; *P* ≤ 0.01) and Dynamic tasks (by 21.1%; *d *=* *0.8; *P* ≤ 0.01). The PST and PST_PA_ tests showed no significant difference between‐ or within‐groups. See Table 3.

**Table 4:** Baseline and postintervention values for the mini‐BEST and its four domains of balance

Outcomes	IMT (*n* = 23)			Sham‐IMT (*n* = 23)			*P*‐values
	Baseline	Postintervention	% Change	Baseline	Postintervention	% Change	Between‐groups
Mini‐BEST	20.4 ± 3.5	24.1 ± 2.2**	18.1	20.8 ± 3.3	21.3 ± 2.9	2.4	*P* = 0.05
Anticipatory	5.0 ± 0.9	5.5 ± 0.6**	10.0	5.2 ± 1.2	4.9 ± 0.9	−5.8	NS
Reactive	3.3 ± 1.7	4.6 ± 1.2**	39.4	3.0 ± 1.7	4.0 ± 1.3	33.3	NS
Sensory	4.9 ± 0.6	5.4 ± 0.6**	10.2	5.1 ± 0.8	5.1 ± 0.6 0	0	NS
Dynamic	7.1 ± 1.5	8.6 ± 0.9**	21.1	7.3 ± 1.6	7.3 ± 1.6 0	0	NS

The mini‐BEST test has a maximum score (MS) of 28, and it is composed of four component Anticipatory MS 6; Reactive postural control MS 6; Sensory orientation MS 6; Dynamic gait MS 10. ^**^significantly different from baseline (*P* ≤ 0.01) NS = not significant.

### Trunk muscle strength

There were significant between‐group differences in anterior (sit‐up, *P* = 0.02) and posterior (Biering–Sørensen, *P* ≤ 0.01) trunk endurance tests (Table 3). Within‐group analysis showed that the IMT group exhibited significant improvements in the Biering–Sørensen test performance (by 63%; *d *=* *3.7; *P* < 0.01).

## Discussion

This is the first study to investigate the effects of home‐based, unsupervised IMT on measures of balance and physical performance in community‐dwelling, older adults. Using a double‐blind, placebo controlled‐design, our findings support the study hypothesis that IMT enhances dynamic balance performance, as evidenced by improvements in the mini‐BEST test performance. In addition, we have found that healthy older adults can undertake IMT safely at home, unsupervised for 8 weeks. The drop‐out rate was 24% for IMT and 21% for sham‐IMT particularly given that (1) motivational calls were not provided and, (2) participants performed the exercises unsupervised (only five out of 30 participants were unable to follow IMT) during the study, due to a reason not related to IMT.

### Effects of IMT on pulmonary and inspiratory muscle function

Pulmonary function (FVC and FEV_1_) remained unchanged after 8 weeks of training in both groups. In accordance with Mills et al. (2015), we propose that improvements in spirometry measurements following IMT are mostly related to task‐learning effects, rather than physiological improvements. Following IMT, inspiratory muscle shortening velocity (PIFR) moderately increased (by 19.7%), whereas sham‐IMT increased to a lesser extent (by 7.5%). These findings are similar to those observed by Mills and colleagues (2015) with healthy older adults (68 ± 3 years). Our participants improved inspiratory muscle strength (MIP) significantly following IMT, which supports previous research in older women (aged 68 ± 5 years) (Souza et al. 2014). However, we also observed improvements in MIP following sham‐IMT that are similar to those reported for older (70 ± 8 years old) heart failure patients (Bosnak‐Guclu et al. 2011) and with young (18 ± 2 years old) elite swimmers (Mickleborough et al. 2008).

The improvements in MIP and PIFR following sham training may reflect enhanced neural activation, rather than respiratory muscle remodeling per se (Eastwood et al. 1998). Accordingly, we must conclude that at least part of the improvements in MIP observed in the IMT group, also reflects an improvement in neural activation. The inspiratory peak power generated during inspiratory loading (Table 2 and Fig. 2) assesses the ability of the inspiratory muscles to shorten under different loads. A greater flow at any given load is indicative of a higher inspiratory power output resulting in faster muscle shortening. Following 8 weeks of IMT, participants significantly improved the peak power at all loads, with the greatest improvement at 80% of their MIP (peak power increased by 80.6% or 2.9 W).

Conversely, in the sham‐IMT group, participants showed significant improvement only at 80% of MIP (peak power increased by 16.3% or 0.7 W). Our results concur with those observed by Romer and McConnell (2003) with young adults (25 ± 2.8 years). Overall, our findings support the effectiveness of IMT, which can be undertaken by community‐dwelling older adults in their own homes without supervision, to enhance pulmonary and inspiratory muscle function. For future research, we recommend decreasing or removing the loading resistance to reduce potential task‐learning effects, for control groups.

### Effects of IMT on physical performance

Following both interventions, the FSTS did not improve, but the sham‐IMT group showed a significant decrease in FSTS_PA_ performance (−3.2 sec) from baseline. This may be attributed to impairment in postural control caused by inspiratory muscle fatigue. In addition, the FSTS has deemed a reasonable choice for clinical and research assessments (Zhang et al. 2018). However, it may not be an ideal test to address changes induced by IMT. Firstly, because the FSTS results mostly correlate to quadriceps strength (Lord et al. 2002), thus potentially the test is insensitive to changes in the trunk musculoskeletal system.

Secondly, the FSTS presents a floor effect that can reduce the validity of the measurement in older adults (Guralnik et al. 1994). For further investigation, we recommended using variants of the sit‐to‐stand test that presents a lower ceiling effect and can produce more stress to the musculoskeletal system (e.g., 30 sec sit‐to‐stand). After 8 weeks the IMT group was able to complete the TUG significantly faster (−5.3%) compared to baseline. Analysis between single and dual tasks revealed that the dual tasks (TUGc and TUG_M_) were significantly different from the single task (TUG), in both groups at baseline and postintervention. However, only in the sham‐IMT group did the TUG_C_ and the TUG_M_ differ significantly from each other postintervention (by 1.7 sec). These changes show that the sham‐IMT group experienced greater difficulty in the cognitive dual task than in the motor dual task, when compared to IMT group. This may indicate a possible decrement in gait ability for the sham‐IMT group. These findings are in accordance with reported amelioration in walking ability, measured through the shuttle walking test, with patients with COPD following IMT (Elmorsi et al. 2016).

Our results on the TUG test are also similar, in magnitude, from those observed following 12 weeks of OTAGO exercise program with 27 healthy home resident older adults (aged 78 ± 8 years) (Kocic et al. 2018). However, it is not possible to conclude what mechanism(s) contributed to the improvements in gait proficiency. We believe that an increase in gait ability can be the result of either: improved VO_2_ uptake, that reduced the feeling of fatigue in the lower limbs (Bailey et al. 2010), or an increase in inspiratory muscle strength, that produced enforcements to the upper and lower body segment linkage, similar to that observed in other trunk muscle exercises (Granacher et al. 2013). Further investigation is required to understand the potential mechanism(s) by which IMT may enhance walking ability in older adults.

### Effects of IMT on balance performance

This is the first study to describe the effects of IMT upon balance performance. The mini‐BEST score improved significantly following IMT (18.1%), whilst sham‐IMT did not differ significantly. Our results are similar in magnitude to those seen after 12 weeks of therapeutic yoga, with community‐dwelling older adults (Kelley et al. 2014). Subsequent analysis of the different mini‐BEST's components showed that the greatest improvements were in reactive (by 39.4%) and dynamic (by 21.1%) tasks, Table 4.

Hodges and colleagues Hodges and Gandevia (2000) established that diaphragm phasic contractions assist in maintaining postural stability in situations whereby external forces (i.e., rapid movement of upper limb) destabilize the spine. We believe that a similar mechanism occurs during reactive (i.e., compensatory stepping correction) and dynamic (e.g., walking at different speeds) tasks, and that improved inspiratory muscle strength resulted in a subsequent improvement in dynamic balance abilities. The positive changes may also be related to the participants' ability to increase intraabdominal pressure that supports the spine (i.e., Valsalva maneuvre) (Hodges et al. 2005).

The absence of significant influence of IMT on PST is likely to relate with the specific postural challenges created by the PST (i.e., standing blindfolded on a stable surface) do not require recruitment of the trunk musculature. Furthermore, it appears that an ankle‐driven strategy (as opposed to hip strategy) dominates the compensatory response to balance perturbation in similar tasks (Horak 1987). In addition, the improvements in balance observed for the IMT group (measured with the mini‐BEST) were greater than those observed following 12 weeks of OTAGO exercise training (measured with Berg Balance scale) for healthy older adults (n = 27; aged 78 ± 8 years; IMT *d *=* *1.3; OTAGO *d *=* *0.6) (Kocic et al. 2018). We can conclude that following 8 weeks of IMT, participants increased balance performance, particularly in dynamic balance, which is relevant for falls risk. Balance improvements may be related to positive changes in inspiratory muscle strength (measured with MIP, PIFR and PP) that caused positive changes in diaphragmatic phasic contractions, and in the ability to increase intraabdominal pressure. We recommend further investigation in the possible mechanism(s) by which IMT improves balance proficiency.

### Effects of IMT on trunk muscle endurance

The posterior trunk muscle endurance tests (i.e., Biering–Sørensen test) were completed by 39 participants out of 46. The reason because seven participants did not perform the assessment was due to discomfort while performing it. Participants in the IMT group showed significant improvements (by 63%) in the posterior trunk endurance test, whilst the sham‐IMT group was no different after 8 weeks. Our findings agree with previous research reporting that IMT enhances endurance plank performance in young recreational runners (Tong et al. 2016). However, it remains unclear as to how IMT may mechanistically contribute to improved trunk muscle endurance for older adults.

### Effects of preactivation of the inspiratory muscles

Studies with young participants (*˜*20 years) reported that preactivation of the inspiratory muscles significantly improves rowing performance (Volianitis et al. 2001). Volianitis et al. (1999) concluded that a “warm‐up” phenomenon, similar to the one present in locomotory muscles, occurs for the inspiratory muscles. Our results support Voliantis' supposition of a respiratory “warm‐up” effect, since both our groups improved FSTS_PA_ time to a greater extent than FSTS (IMT by 10.7%; sham‐IMT by 10.0%). We also noticed that the IMT group had minor changes between baseline and postintervention in FSTS_PA,_ whilst the sham‐IMT group showed significant changes, needing more time to complete the FSTS_PA_ (by 20.5%). These results indicate that combining IMT and inspiratory muscle preactivation can lead to improvements in FSTS tasks. We can then conclude that a “warm‐up” effect of inspiratory muscles can improve physical performance outcomes and that IMT can help in maintaining this performance in older adults.

## Conclusion

Using a double‐blind, randomized placebo‐controlled design, we investigated the effectiveness of 8 weeks of unsupervised, home‐based IMT with healthy older adults. After 8 weeks the IMT group showed improvements in inspiratory muscle function (PIFR, MIP and peak inspiratory power), as well as dynamic balance ability, as shown by increments in the mini‐BEST score. The study also showed that IMT is feasible and effective for healthy older adults when delivered at home and unsupervised, without adverse events, such as inducing falls accident, or discomfort.

Inspiratory muscle function improvements in the placebo group suggest that compared to younger adults, healthy older adults may rely more greatly on neural systems. Further research is required to determine the potential mechanism(s) by which inspiratory muscles contribute to dynamic and static balance, as well as the role that IMT may play in reducing the risk of falling compared to other balance interventions (e.g., OTAGO exercise program).

## Limitations and Recommendations

The main limitation of this study was that PST may have been insensitive to detecting changes elicited by IMT. We believe that, in the absence of external perturbation, participants used primarily an ankle‐driven strategy as opposed to a hip strategy (Horak 1987). The ankle strategy has been shown to be adopted for more than 90% of the time to cope with task demand in similar assessments for young adults (23 ± 3.6 years) (Blenkinsop et al. 2017). Therefore, without the necessary hip and trunk muscle recruitment, the effect of IMT on balance was not detectable. Future research should investigate the role that IMT may play in increasing balance proficiency for frailer adults (i.e., care home residents).

Inspiratory muscle adaptations to training exhibit the specificity principle, i.e., high‐load training increases maximal static pressures and high‐flow training increase maximal inspiratory flow rate Romer and McConnell 2003. Future studies should explore the effects of different IMT protocols upon balance outcomes (e.g., high load vs. high flow).

Future research that aims to investigate the relationship between improvements in inspiratory muscle function and balance proficiency should also consider the inclusion of additional assessments to measure diaphragm thickness (e.g., ultrasonography). These measurements might shed light on potential mechanisms.

## Conflict of Interest

This work was sponsored by Bournemouth University. FF, JG and TW declare no conflicts of interests. AM acknowledges a previous (now expired) beneficial interest in POWERbreathe^®^ inspiratory muscle trainers in the form of a share of royalty income to the University of Birmingham and a potential share of royalty income to Brunel University. In the past, AM has also provided consultancy services to POWERbreathe^®^ International Ltd., but no longer does so. AM is named on two patents relating to POWERbreathe^®^ products, including the device used in the present study, as well as being the author of two books on inspiratory muscle training.

## References

[phy214076-bib-0001] Allet, L. , S. Armand , A. Golay , D. Monnin , de Bie R. A. , and de Bruin E. D. . 2008 Gait characteristics of diabetic patients: a systematic review. John Wiley & Sons Ltd, Great Britain p. 173. 10.1002/dmrr.809 18232063

[phy214076-bib-0002] Atkinson, G. , and T. Reilly . 1996 Circadian variation in sports performance. Sports Med. (Auckland, NZ) 21:292–312. 10.2165/00007256-199621040-00005 8726347

[phy214076-bib-0003] Bailey, S. J. , L. M. Romer , J. Kelly , D. P. Wilkerson , F. J. DiMenna , and A. M. Jones . 2010 Inspiratory muscle training enhances pulmonary O_2_ uptake kinetics and high‐intensity exercise tolerance in humans. J. Appl. Physiol. 109:457–468. 20507969 10.1152/japplphysiol.00077.2010

[phy214076-bib-0004] Biering‐Sørensen, F. 1984 Physical measurements as risk indicators for low‐back trouble over a one‐year period. Spine 9:106–119. 6233709 10.1097/00007632-198403000-00002

[phy214076-bib-0005] Blenkinsop, G. M. , M. T. G. Pain , and M. J. Hiley . 2017 Balance control strategies during perturbed and unperturbed balance in standing and handstand. Roy. Soc. Open Sci. 4:161018. 28791131 10.1098/rsos.161018 PMC5541526

[phy214076-bib-0006] Bosnak‐Guclu, M. , H. Arikan , S. Savci , D. Inal‐Ince , E. Tulumen , K. Aytemir , et al. 2011 Effects of inspiratory muscle training in patients with heart failure. Elsevier Science B.V., Amsterdam, Great Britain p. 1671. 10.1016/j.rmed.2011.05.001 21621993

[phy214076-bib-0007] Britto, R. R. , C. C. Zampa , T. A. de Oliveira , L. F. Prado , and V. F. Parreira . 2009 Effects of the aging process on respiratory function. Gerontology 55:505–510 506p. 19713688 10.1159/000235853

[phy214076-bib-0008] CDCP . 2017 Centers for Disease Control and Prevention. Web‐based injury statistics query and reporting system (WISQARS). National Center for Injury Prevention and Control, Centers for Disease Control and Prevention. (Available at http://www.cdc.gov/injury/wisqars/index.html (accessed May 2017).

[phy214076-bib-0009] Charususin, N. , R. Gosselink , M. Decramer , H. Demeyer , A. McConnell , D. Saey , et al. 2018 Randomised controlled trial of adjunctive inspiratory muscle training for patients with COPD. Thorax 3:942–950. 10.1136/thoraxjnl-2017-211417 29914940

[phy214076-bib-0010] Eastwood, P. R. , D. R. Hillman , A. R. Morton , and K. E. Finucane . 1998 The effects of learning on the ventilatory responses to inspiratory threshold loading. Am. J. Respir. Crit. Care Med. 158:1190–1196. 9769281 10.1164/ajrccm.158.4.9803108

[phy214076-bib-0011] Elmorsi, A. S. , M. E. Eldesoky , M. A. A. Mohsen , N. M. Shalaby , and D. A. Abdalla . 2016 Effect of inspiratory muscle training on exercise performance and quality of life in patients with chronic obstructive pulmonary disease. Egypt. J. Chest Dis. Tuberc. 65:41–46.

[phy214076-bib-0012] Enright, P. L. , R. A. Kronmal , T. A. Manolio , M. B. Schenker , and R. E. Hyatt . 1994 Respiratory muscle strength in the elderly. Am. J. Respir. Crit. Care Med. 149:430–438. 8306041 10.1164/ajrccm.149.2.8306041

[phy214076-bib-0013] Fairbank, J. C. T. , and P. B. Pynsent . 2000 The Oswestry Disability Index…with commentary by Walsh T. Spine 25:2940–2953. 11074683 10.1097/00007632-200011150-00017

[phy214076-bib-0014] Faul, F. , E. Erdfelder , A.‐G. Lang , and A. Buchner . 2007 G*Power 3: a flexible statistical power analysis program for the social, behavioural, and biomedical sciences. Behav. Res. Methods 39:175–191. 17695343 10.3758/bf03193146

[phy214076-bib-0015] Folstein, M. F. , L. N. Robins , and J. E. Helzer . 1983 The mini‐mental state examination. Archiv. Gen. Psychiatr. 40:812. 10.1001/archpsyc.1983.01790060110016 6860082

[phy214076-bib-0016] Granacher, U. , A. Gollhofer , T. Hortobagyi , R. W. Kressig , and T. Muehlbauer . 2013 The importance of trunk muscle strength for balance, functional performance, and fall prevention in seniors: a systematic review. Sports Med. 43:627–641. 23568373 10.1007/s40279-013-0041-1

[phy214076-bib-0017] Guralnik, J. M. , E. M. Simonsick , L. Ferrucci , R. J. Glynn , L. F. Berkman , D. G. Blazer , et al. 1994 A short physical performance battery assessing lower extremity function: association with self‐reported disability and prediction of mortality and nursing home admission. J. Gerontol. 49:M85–M94. 8126356 10.1093/geronj/49.2.m85

[phy214076-bib-0018] Hodges, P. W. , and S. C. Gandevia . 2000 Activation of the human diaphragm during a repetitive postural task. J. Physiol. 522(Pt 1):165–175. 10618161 10.1111/j.1469-7793.2000.t01-1-00165.xm PMC2269747

[phy214076-bib-0019] Hodges, P. W. , A. E. Eriksson , D. Shirley , and S. C. Gandevia . 2005 Intra‐abdominal pressure increases stiffness of the lumbar spine. J. Biomech. 38:1873–1880. 16023475 10.1016/j.jbiomech.2004.08.016

[phy214076-bib-0020] Horak, F. B. 1987 Clinical measurement of postural control in adults. Phys. Ther. 67:1881–1885. 3685116 10.1093/ptj/67.12.1881

[phy214076-bib-0021] Janssens, L. , S. Brumagne , A. K. McConnell , K. Claeys , M. Pijnenburg , N. Goossens , et al. 2014 Impaired postural control reduces sit‐to‐stand‐to‐sit performance in individuals with chronic obstructive pulmonary disease. PLoS ONE 9:e88247. 24533072 10.1371/journal.pone.0088247 PMC3922802

[phy214076-bib-0022] Kelley, K. K. , D. Aaron , K. Hynds , E. Machado , and M. Wolff . 2014 The effects of a therapeutic yoga program on postural control, mobility, and gait speed in community‐dwelling older adults. J. Altern. Complement. Med. 20:949–954. 25148571 10.1089/acm.2014.0156 PMC4270164

[phy214076-bib-0023] Kocic, M. , Z. Stojanovic , D. Nikolic , M. Lazovic , R. Grbic , L. Dimitrijevic , et al. 2018 The effectiveness of group Otago exercise program on physical function in nursing home residents older than 65 years: a randomized controlled trial. Arch. Gerontol. Geriatr. 75:112–118. 29241091 10.1016/j.archger.2017.12.001

[phy214076-bib-0024] Langer, D. , C. Jacome , N. Charususin , H. Scheers , A. McConnell , M. Decramer , et al. 2013 Measurement validity of an electronic inspiratory loading device during a loaded breathing task in patients with COPD. Elsevier Science B.V., Amsterdam, Great Britain p. 633. 10.1016/j.rmed.2013.01.020 23421970

[phy214076-bib-0025] Lord, S. R. , S. M. Murray , K. Chapman , B. Munro , and A. Tiedemann . 2002 Sit‐to‐stand performance depends on sensation, speed, balance, and psychological status in addition to strength in older people. J. Gerontol. A Biol. Sci. Med. Sci. 57:M539–M543. 12145369 10.1093/gerona/57.8.m539

[phy214076-bib-0026] Martin, M. 2010 Structural and physiological age‐associated changes in aging lungs. Semin. Respir. Crit. Care Med. 31:521–527. 20941653 10.1055/s-0030-1265893

[phy214076-bib-0027] McConnell, A. 2013 Respiratory muscle training : theory and practice. Churchill Livingstone, Oxford.

[phy214076-bib-0028] McGill, S. M. , A. Childs , and C. Liebenson . 1999 Endurance times for low back stabilization exercises: clinical targets for testing and training from a normal database. Arch. Phys. Med. Rehabil. 80:941–944. 10453772 10.1016/s0003-9993(99)90087-4

[phy214076-bib-0029] Mickleborough, T. D. , J. M. Stager , K. Chatham , M. R. Lindley , and A. A. Ionescu . 2008 Pulmonary adaptations to swim and inspiratory muscle training. Eur. J. Appl. Physiol. 103:635–646. 18478253 10.1007/s00421-008-0759-x

[phy214076-bib-0030] Miller, M. R. , J. Hankinson , V. Brusasco , F. Burgos , R. Casaburi , A. Coates , et al. 2005 Standardisation of spirometry. Eur. Respir. J. 26:319–338. 16055882 10.1183/09031936.05.00034805

[phy214076-bib-0031] Mills, D. E. , M. A. Johnson , Y. A. Barnett , W. H. T. Smith , and G. R. Sharpe . 2015 The effects of inspiratory muscle training in older adults. Med. Sci. Sports Exerc. 47:691–697. 25116085 10.1249/MSS.0000000000000474

[phy214076-bib-0032] Mourey, F. , A. Grishin , P. d'Athis , T. Pozzo , and P. Stapley . 2000 Standing up from a chair as a dynamic equilibrium task: a comparison between young and elderly subjects. J. Gerontol. A Biol. Sci. Med. Sci. 55:B425–B431. 10995039 10.1093/gerona/55.9.b425

[phy214076-bib-0033] NICE ICGP . 2013 Falls in older people: assessing risk and prevention. 39960998

[phy214076-bib-0034] O'Hoski, S. , K. M. Sibley , D. Brooks , and M. K. Beauchamp . 2015 Construct validity of the BESTest, mini‐BESTest and briefBESTest in adults aged 50 years and older. Gait Posture. 42:301–305. 26183191 10.1016/j.gaitpost.2015.06.006

[phy214076-bib-0035] Parraca, J. A. , P. R. Olivares , A. N. A. Carbonell‐Baeza , V. A. Aparicio , J. C. Adsuar , and N. Gusi . 2011 Test‐Retest reliability of Biodex Balance SD on physically active old people. J. Hum. Sport Exerc. 6:444.

[phy214076-bib-0036] Podsiadlo, D. , and S. Richardson . 1991 The timed ‘Up and Go': a test of basic functional mobility for frail elderly persons. J. Am. Geriatr. Soc. 39:142–148. 1991946 10.1111/j.1532-5415.1991.tb01616.x

[phy214076-bib-0037] Powell, L. E. , and A. M. Myers . 1995 The activities‐specific balance confidence (ABC) scale. J. Gerontol. A Biol. Sci. Med. Sci. 50A:M28–M34. 7814786 10.1093/gerona/50a.1.m28

[phy214076-bib-0038] Romer, L. M. , and A. K. McConnell . 2003 Specificity and reversibility of inspiratory muscle training. Med. Sci. Sports Exerc. 35:237–244. 12569211 10.1249/01.MSS.0000048642.58419.1E

[phy214076-bib-0039] Romer, L. M. , A. K. McConnell , and D. A. Jones . 2002 Effects of inspiratory muscle training on time‐trial performance in trained cyclists. J. Sports Sci. 20:547–562. 12166881 10.1080/026404102760000053

[phy214076-bib-0040] Sherrington, C. , N. J. Fairhall , G. K. Wallbank , A. Tiedemann , Z. A. Michaleff , K. Howard , L. Clemson , S. Hopewell , and S. E. Lamb 2019 Exercise for preventing falls in older people living in the community. Cochrane Database of Systematic Reviews 1:CD012424 DOI: 10.1002/14651858.CD012424.pub2 30703272 10.1002/14651858.CD012424.pub2 PMC6360922

[phy214076-bib-0041] Souza, H. , T. Rocha , M. Pessoa , C. Rattes , D. Brandao , G. Fregonezi , et al. 2014 Effects of inspiratory muscle training in elderly women on respiratory muscle strength, diaphragm thickness and mobility. J. Gerontol. Series 69:1545–1553. 10.1093/gerona/glu182 25395284

[phy214076-bib-0042] Tong, T. K. , A. K. McConnell , H. Lin , J. Nie , H. Zhang , and J. Wang . 2016 ‘Functional' inspiratory and core muscle training enhances running performance and economy. J. Strength Cond. Res. 30:2942–2951. 25162653 10.1519/JSC.0000000000000656

[phy214076-bib-0043] Volianitis, S. , A. K. McConnell , Y. Koutedakis , and D. A. Jones . 1999 The influence of prior activity upon inspiratory muscle strength in rowers and non‐rowers. Int. J. Sports Med. 20:542–547. 10606219 10.1055/s-1999-9464

[phy214076-bib-0044] Volianitis, S. , A. K. McConnell , Y. Koutedakis , and D. A. Jones . 2001 Specific respiratory warm‐up improves rowing performance and exertional dyspnea. Med. Sci. Sports Exerc. 33:1189–1193. 11445767 10.1097/00005768-200107000-00017

[phy214076-bib-0045] Watt, A. , C. J. Clark , and J. M. Williams . 2018 Differences in sit‐to‐stand, standing sway and stairs between community‐dwelling fallers and non‐fallers: a review of the literature(In press).

[phy214076-bib-0046] Zhang, Q. , Y.‐X. Li , X.‐L. Li , Y. Yin , R.‐L. Li , X. Qiao X , et al. 2018 A comparative study of the five‐repetition sit‐to‐stand test and the 30‐second sit‐to‐stand test to assess exercise tolerance in COPD patients. Int. J. Chron. Obstruct. Pulmon. Dis. 13:2833. 30237707 10.2147/COPD.S173509 PMC6136403

